# Hybrid management of iliac injury during thoracic endovascular aortic repair: A case report

**DOI:** 10.1177/2050313X241236328

**Published:** 2024-05-22

**Authors:** Pierfrancesco Antonio Annuvolo, Enrico Centritto, Veronica Picone, Maurizio Maiorano, Maria Giannantonio, Pietro Modugno

**Affiliations:** 1 Unit of Vascular Surgery, Fondazione Policlinico Universitario Gemelli IRCCS—Università Cattolica del Sacro Cuore, Rome, Italy; 2 Unit of Vascular Surgery, Responsible Research Hospital, Campobasso, Italy; 3 Anesthesia and Intensive Care Unit, Responsible Research Hospital, Campobasso, Italy

**Keywords:** Iliac, injury, TEVAR, rupture, hybrid

## Abstract

Thoracic endovascular aortic repair is nowadays the preferred option to manage descending thoracic aorta diseases. However, despite feasibility and safety of the procedures, several complications may occur. We report the case of an 83-year-old female patient with inadvertent iliac rupture occurred during thoracic endovascular aortic repair. To limit massive bleeding, considering the patient’s comorbidities contraindicating open surgical repair and the morphology of the arterial injury (circumferential rupture of the artery from its origin), we chose to perform a homolateral hypogastric and common iliac artery embolization and an aorto-uniliac balloon expandable stent graft deployment from the distal aorta to the contralateral common iliac artery. A femoro-femoral crossover bypass graft was performed to restore both lower limbs perfusion. Final angiography documented correct positioning and regular patency of the implanted grafts and bypass with no blood loss from the right iliac vessels. Despite careful preoperative assessment, iliac artery injury can represent a challenging complication of thoracic endovascular aortic repair, particularly in the setting of inadequate iliac diameter, calcification and vessel tortuosity, or when large-caliber introducers are required. The hybrid approach we describe is a valid and effective solution to minimize blood loss and avoid major consequences in the management of iatrogenic iliac artery rupture during endovascular procedures.

## Introduction

Endovascular repair techniques for thoracic and abdominal aneurysms are increasingly popular today because of their excellent medium- to long-term results and low peri- and post-operative complication rates. Thoracic endovascular aortic repair (TEVAR) is nowadays the preferred option to manage descending thoracic aorta diseases.^1^

A proper pre-operative planning regarding patient’s anatomical features is necessary for successful intervention. Inadequate accesses due to small size or extreme tortuosity of iliac arteries can be considered an exclusion criterion for an endovascular procedure.^2^ In up to 47% of the cases, endovascular aortic repair is challenged by significant underlying iliac arterial disease and anatomy.^3^ These can lead to several complications,^4^ such as difficult positioning of the endograft, that can require a variety of supplemental surgical and endovascular procedures to facilitate endograft placement, or even iatrogenic iliac arterial rupture, that presents a life-threatening condition and a therapeutic challenge for the operating surgeons.^5^

However, these percentages are declining in recent years with improvements in technology of new generation endografts, in terms of lower profile and greater trackability.

When an inadvertent iliac rupture occurred, open surgical or endovascular bail-out techniques can be afforded to avoid major bleeding and hemodynamic failure.

Another less common but valid approach can be represented by the hybrid technique, often described in literature as an alternative option to surgery for aorto-iliac aneurysms repair.^6,7^

In our case of an inadvertent iliac rupture occurred during TEVAR, we described how we managed this potentially fatal situation with an uncommon hybrid approach.

This case is reported in line with the Surgical CAse REport criteria.^8^

## Case presentation

**Figure 1.**
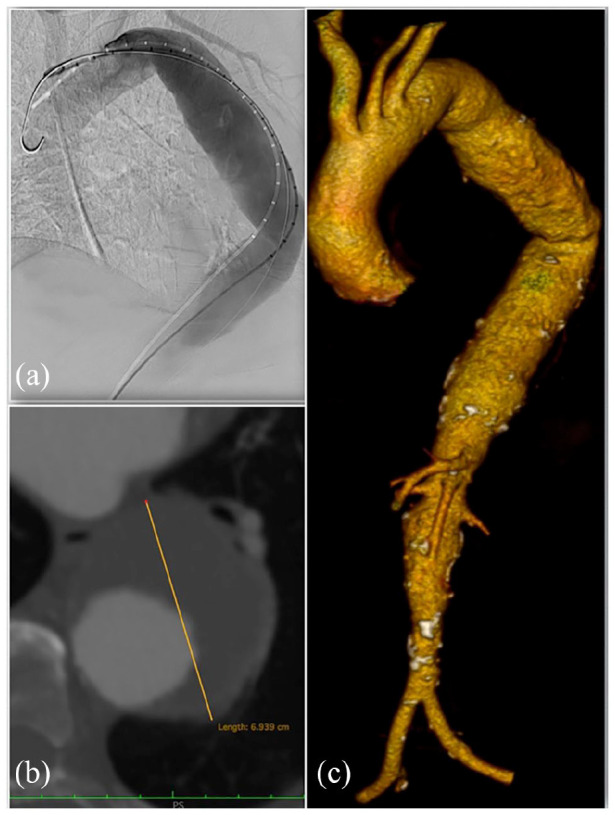
Pre-operative images showing the descending thoracic aorta aneurysm; digital subtraction angiography (a); axial plane (b); and 3D volume rendering (c).

An 83-year-old female patient was admitted to our Institution with an occasional finding of a descending thoracic aortic aneurysm with a maximum diameter of 70 mm on computed tomography angiography (CTA) (Figure 1). She had a history of smoking habit, hypertension and hyperuricemia, and no previous surgery. She was on single antiplatelet therapy.

She underwent a TEVAR. Before operation, femoral vessels were assessed and normal diameters were found (maximum diameter of common femoral artery 8.5 mm bilaterally, no calcifications).

Through a right common femoral artery surgical access, a E-Vita 3G (JOTEC—Anderstorp, SE) thoracic endograft was deployed in the thoracic aorta. The 24 Fr graft’s introducer sheath was replaced with a Sentrant 22 Fr introducer sheath (Medtronic Inc.—Minneapolis, USA) placed in the right iliac axis. Angiography was performed to demonstrate correct graft positioning with complete exclusion of the aneurysm and no endoleaks.

**Figure 2.**
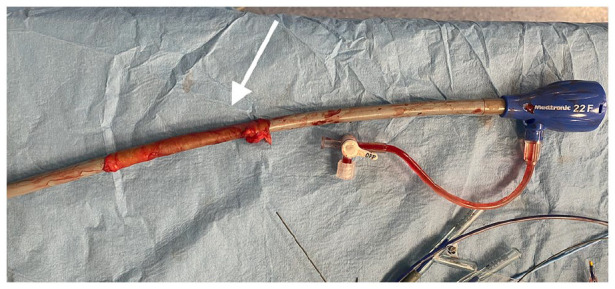
External iliac artery completely dislodged from its origin still on the Medtronic Sentrant 22 Fr introducer sheath.

**Figure 3.**
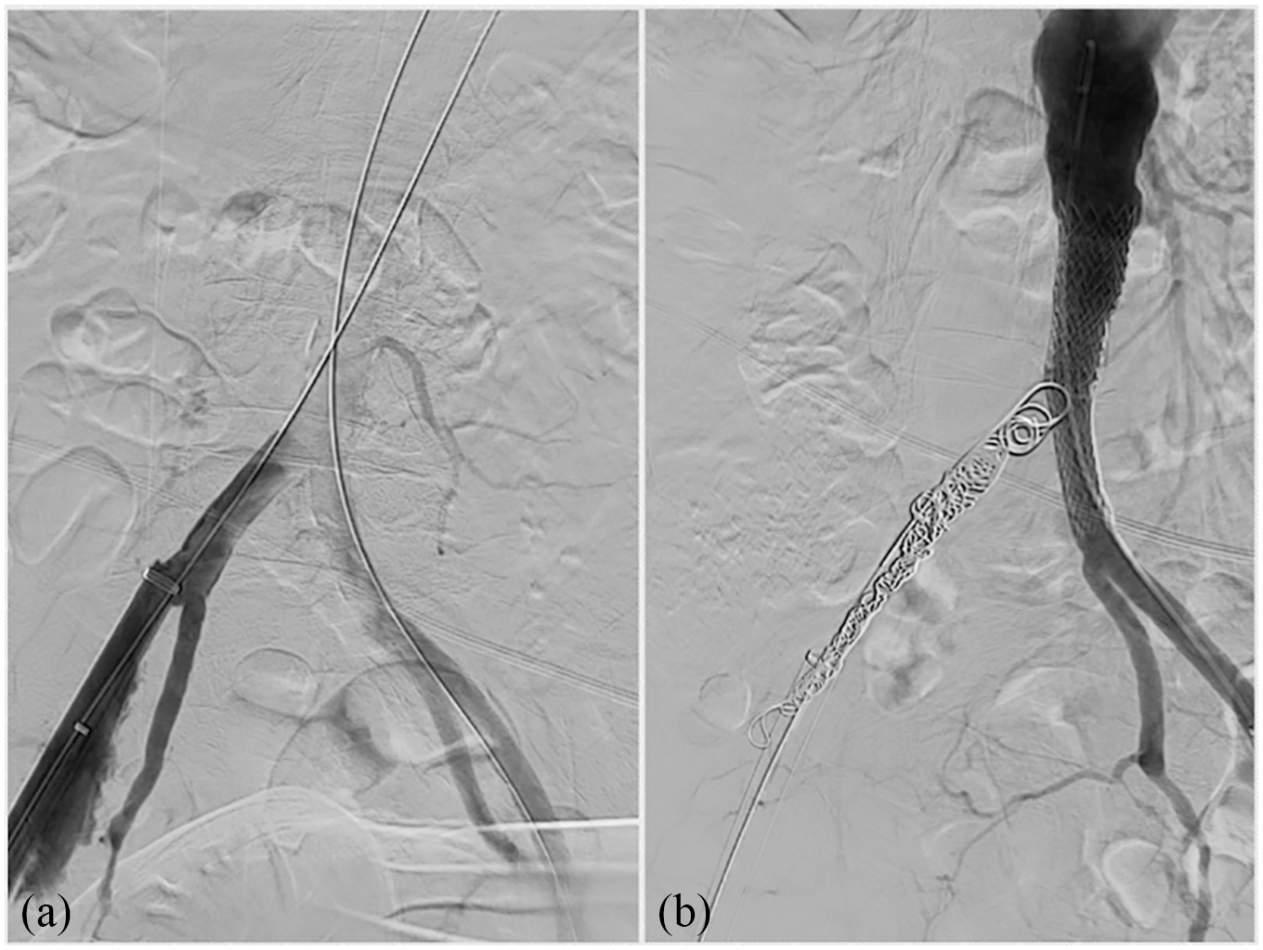
Angiography showing massive bleeding at the right iliac fracture site (a); aorto-iliac endovascular reconstruction with balloon expandable stent graft and coils (b).

During sheath removal, a circumferential rupture of the right external iliac artery (EIA) occurred at the level of the common iliac artery bifurcation. (Figures 2–3(a)) In order to limit the massive bleeding, an aortic occlusion balloon (Giant-S, SciTech—Aparecida de Goiania, GO) was placed in the distal aorta. The right hypogastric and common iliac arteries were embolized through the contralateral femoral access with multiple pushable 0.018 fibered platinum coils (Complex Helical, Boston Scientific—Marlborough, USA and Nester, Cook—Bloomington, USA). An aorto-uniliac balloon expandable stent graft (Bentley BeGraft 20 × 48 mm and 14 × 59 mm, InnoMed—Hechingen, DE) was deployed from the distal aorta to the left common iliac artery through a left common femoral artery surgical access (Figure 3(b)).

To maintain perfusion of both lower limbs, a left-to-right femoro-femoral crossover bypass was performed with an 8 mm heparinized polytetrafluoroethylene graft (Propaten, W. L. Gore—Newark, USA). The common femoral artery was ligated to prevent retrograde bleeding.

Final angiography documented correct positioning of the thoracic endoprosthesis, regular patency of the left aorto-uniliac stent graft and of the femoro-femoral bypass with no blood loss from the right iliac arteries.

The post-operative course was uneventful except for mild left arm weakness on post-operative day 2.

CT scan of the brain showed no evidence of ischemia or hemorrhage. Brain and spinal cord MRI were performed without evidence of spinal cord ischemia. However, the patient underwent neurological consultation and rehabilitation with complete regression of the impairment.

**Figure 4.**
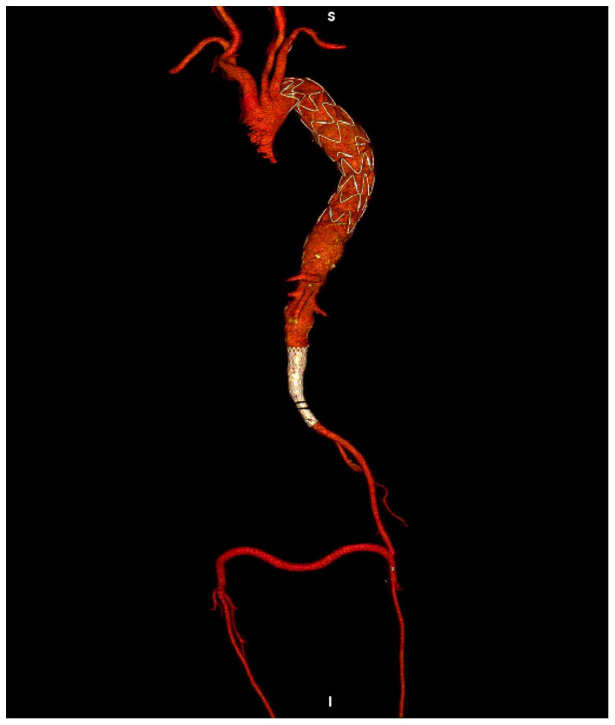
Post-operative CTA demonstrating correct positioning of the stent grafts and regular patency of the bypass.

A 1-week post-operative CTA showed correct positioning of the thoracic endoprosthesis and of the left aorto-uniliac stent graft and regular patency of the femoro-femoral bypass (Figure 4).

The patient was discharged home on post-operative day 14 on single antiplatelet therapy. She was in good clinical condition with no neurological impairment.

**Figure 5.**
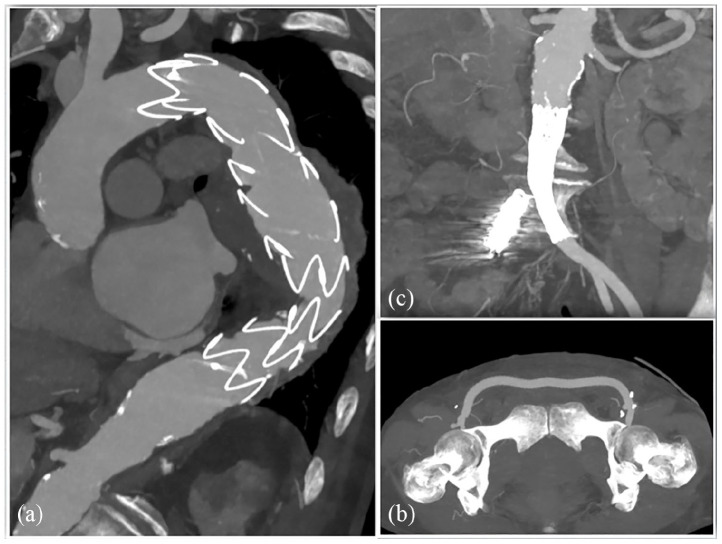
Six months follow-up CTA showing correct aneurysm exclusion (a); regular patency of the stent grafts and bypass (b and c) and no endoleaks.

The 6-month follow-up CTA showed correct aneurysm exclusion and no endoleaks (Figure 5). Physical examination revealed a good clinical condition with optimal perfusion of both lower limbs.

## Discussions

One of the most important factors to consider in the pre-operative planning of an endovascular aortic repair is an adequate vascular access through the femoral and iliac arteries.

Calcifications, small sizes and tortuosity of the vessels have been associated with an increased incidence of iliac injuries.^9^ These lesions are more common in thoracic than in abdominal endografting because of the higher percentage of women treated and the larger graft sizes used.^10^

The exponential increase of endovascular treatments and the associated use of large sheaths and delivery devices may cause iliac vessels to be damaged by traction, even without adverse anatomical features, as it happened in our case.

Before TEVAR procedure, we performed a proper pre-operative CT scan assessment. No problems were found in the iliac accesses in terms of calcifications, size or tortuosity. Probably the use of a large 22 Fr introducer or the need to maintain it inside the iliac axis for a long time may have led to the terrifying “iliac on a stick.”

Endovascular repair is the most widely used approach to manage this catastrophic event.^11^ In a recent study, Zambetti et al.^12^ identified 507 patients treated for iliac artery injuries and described how, particularly in patients with blunt injury, endovascular repair was associated with lower morbidity (29.3% vs 41.3%; *p* = 0.082) and significantly reduced mortality (14.6% vs 26.7%; *p* = 0.037) compared with the open-repair.

A successful and timely approach requires several techniques such as intraoperative invasive arterial blood pressure monitoring, maintenance of stiff wire access, readily available intra-aortic occlusion balloons for bleeding control, and an inventory of readily available covered stent grafts.^13,14^

During our procedure, the injury occurred by a circumferential rupture of the EIA from its origin and removal of the introducer sheath without removing the vessel itself was not possible. Another treatment option for the patient could have been an endo reconstruction with deployment of a covered stent from the aorta to the right common femoral artery. Unfortunately, an adequately long covered stent was not available at that time. An open repair by laparotomic extraperitoneal access to the iliac arteries was not feasible due to the frailty and age of the patient. So, the decision for a hybrid management through an endovascular exclusion of the ruptured vessel and surgical revascularization of the homolateral lower limb was made.

## Conclusions

Despite careful pre-operative assessment, iliac artery injury can represent a challenging complication of TEVAR, leading to death or major comorbidities. The physician may face this situation particularly in case of inadequate iliac diameter, calcification and tortuosity of the vessel, or when large-caliber introducers are required. The hybrid approach we described in this situation, which is usually used for exclusion of aorto-iliac aneurysms, is a valid and effective solution to minimize blood loss and avoid major consequences in the management of iatrogenic iliac artery rupture during endovascular procedures, especially when a circumferential rupture of an iliac artery has occurred and open repair is not feasible.
